# Fistula*Read before the United States Veterinary Medical Association and International Congress, Chicago, October 18th, 1893.

**Published:** 1893-11

**Authors:** M. H. Reynolds

**Affiliations:** University of Minnesota


### FISTULA.*

By Prof. M. H. Reynolds, M. D., V. M.,
University of Minnesota.

There are few questions in the whole field of veterinary science of which the young practitioner feels that he knows more, and the older wishes he did know more, than those of fistula. Aside from castrations. there is propably no one class of patients with which rural practitioners deal more commonly and none are more disagreeable or more unsatisfactory. We discuss them at every meeting of our State Association, so do all our sister associations, and yet we use about the same old treatment as did our predecessors half a century ago. They are just as dirty and just as disagreeable, just as slow and just as liable to recur.

A thorough discussion of the anatomy concerned in the question of fistula of the Cervical and Anterior Dorsal regions calls for a study of 32 pairs of muscles, 19 pairs of nerves, 1 ligament, and 20 pairs of arteries and their associated veins ; but only the more important can be discussed in a paper of this length.

The muscles are counted as follows : Panniculus Carnosus, 1; all the Superior Cervical, numbering 17; all of the Inferior Cervical, numbering 11; Supra and Infra Spinatus of the External Scapular, and one Internal Scapular, the Subscapularis ; four from the Dorsal region Trapezius, Small Anterior Serratus, Ilio-Spinalis and Common Intercostal. In the Costal region, we will count about four of the External Intercostals. In ordinary practice, however, we rarely find but 17 pairs of muscles associated with this pathological condition.

For brevity's sake, the blood and nerve supply is given with the Myology. An operator, who cares to become an expert in the treatment of these cases. must know where these muscles are, their origin, insertion, direction of fibres, and their function. He must have definite ideas of the location, course, and distribution of the larger blood vessels, and nerves, if he would make very deep incisions and punctures, as he sometimes must do. It is very unpleas-

* Read before the United States Veterinary Medical Association and International Congress, Chicago, October 18th, 1893.

ant to cut the Dorsal, Superior Cervical, or even larger branches of the Vertebral Artery, under several inches of muscular tissue.

The *Panniculus* is a broad sheet of muscular and aponeurotic tissue, somewhat triangular in shape, just under the skin and applied to the sides of thorax and abdomen. The Subcutaneous Thoracic, a branch from the Brachial plexus, with superficial branches of the cervio and dorso-spinal nerves, furnish nerve force. Its blood supply comes through the Dorsal, Vertebral, External Thoracic and Superior Scapular, with all the Dorso-spinal Intercostals.

The *Cervical Ligament*, through its surgical importance, demands more than passing notice. All the Superior Cervical muscles are arranged in layers beneath the Funicular and on either side of the Lamellar portion. The peculiar anatomical features of this great ligament, and its association with related muscles, explain in part the prevalence and persistence of fistulæ in the cervical region. It is easily divided for study into two portions, the funicular and the lamellar. The former is simply a wide band of yellow elastic fibres, with one end inserted in the spinous process of the first dorsal vertebra with the Dorso-lumbar ligament ; the other at the cervical tuberosity of the Occipital bone. Its function is to support the head. The Lamellar consists of two equal plates of similar tissue closely applied, placed vertically in the median line, and filling the triangular space between the funicular portion and the cervical vertebræ. It is attached below to the spinous processes of last six cervical ; to second and third dorsal vertebræ, and above to the funicular. They are in relation externally to one branch of the Ilio Spinal ligament, the Transverse-Spinous muscle of the neck, and Great Complexus. The Superior Cervical muscles, seventeen in number on each side, are arranged in four fairly well defined layers.

The *Trapezius* is a thin triangular muscle, covering the withers and cervical base, attached above to the funicular ligament and transverse processes of the Anterior Dorsal vertebræ, and below by its apical aponeurosis to the Olecranon spine and Scapular aponeurosis. This muscle is covered by two aponeurotic plates, whose fibres run in opposite directions and it, in turn, covers portions of the Rhomboideus splenius, Angularis, Supra and Infra Spinatus. Its blood comes mainly from the Dorsal artery, and its nerve force from the Spinal Accessory. Fibres of the cervical portion run downward and backward.

The *Rhomboideus* has the form of a long triangle-apex forward. It is covered by the Cervical Trapezius, Scapular cartilage, and aponeurosis of the Great Dorsal. It covers the postero-superior, excavated portion of the Splenius, whose fibres it crosses at an acute angle, in running backward and slightly downward. Its origin is on the posterior two-thirds of the cervical ligament, and by fasciculi to the superior spinous processes of the 2d, 3d, 4th and 5th dorsal vertebræ. Its insertion is by the lower extremity to superior and internal surface of scapular cartilage. Its blood supply is from the Dorsal and Superior Cervical; nerve supply is sixth cervical and muscular branch of the Brachial plexus. The anterior fibres run nearly backward and the posterior downward.

*Levator Anguli Scapulæ.* Short, powerful, triangular, thick posterior and inferior borders, thin above, and placed just in front of Scapula. Its origin is on the transverse processes of 3d, 4th, 5th, 6th and 7th Cervical vertebræ by five fasciculi, whose fibres converge to a common tendon which inserts on the anterior triangular surface, internal face of scapula. Externally, in relation with Cervical Trapezius, Mastoido Humeralis and Small Pectoral, and internally with the Splenius, Ilio Spinalis and Common Intercostal. Its fibres all run downward and forward. Blood supply, Dorsal and Superior Cervical; Nerves, Sixth Cervical and muscular branch of the Brachial plexus.

The *Splenius* is one of the larger muscles of this region. Flattened, triangular, bounded above by the cervical cord, below by the first four cervical vertebræ and inferior branch of the Ilio Spinalis, attached by its postero-superior border to the cervical cord and spineous processes of the anterior Dorsal Vertebræ, by its antero-inferior border, to Mastoid crest and transverse processes of first five Cervical vertebræ except the Axis. Externally this muscle is in contact with Rhomboideus, Angularis, Trapezius and Mastoido-humeral. Internally, with both complexus muscles and the Great and Small Oblique muscles of the head. Its fibres run parallel with the Cervical cord. Its nutrition comes through the Dorsal, Superior Cervical and Vertebral arteries. Interesting features of this blood supply are the several anastomoses which the vertebral makes in this region, with the Retrograde branch of the Occipital, with branches of the Superior Cervical, and Occipito Muscular, and the Middle Spinal arteries. The anastomoses are essentially responsible for the persistent hemorrhages which we sometimes get when making incisions in the poll-evil region. The Splenius receives nerve supply through both the Superficial and deep Cervical plexi.

*Great Complexus* a very large, strong muscle, placed between the Splenius and Lamellar portion of the Cervical ligament. It consists of two fairly distinct portions, which lie side by side, and together form an elongated triangle. It covers the Cervical ligament, the superior portion of the Ilio Spinalis, the Transverse Spinous of the neck, the Oblique and Post, Straight muscles of the head, and it in turn is covered by the Splenius and Small Complexus. The anterior portion has, for origin, the transverse processes of 1st and 2d Dorsal and the articular tubercles of the cervical vertebræ. The posterior portion has its origin on the Spinous process of the first, and on transverse processes of first four Dorsal vertebræ. Both portions insert on the Occipital tuberosity, and between them passes the trunk of the Superior Cervical artery. Fibres of the posterior and larger portion run forward and upward nearly parallel with the cervical cord. Those of the anterior portion have a direction more nearly upward, and thus fuse with those of the posterior. Blood supply, Dorsal, Superior cervical and branches from the Deep Cervical plexus.

The *Small Complexus* is also composed of two distinct portions between which, deep seated collections of pus can burrow, and be almost out of surgical reach ; unlike many of the Cervical muscles, it is not triangular but long, almost fusiform. The two portions of this muscle have their fixed insertion in common with the smaller portion of the Great Complexus, on the transverse processes of first two Dorsal and articular tubercles of the Cervical vertebræ. One portion has its fixed insertion on mastoid process of the Temporal bone ; the other on the tranverse process of the Atlas. The Small Complexus is over laid by the Splenius and is related internally to the Great Complexus and the two Oblique muscles. The fibres are parallel with the Cervical cord. Its nourishment comes through the same arteries and it receives nerve force from the same Cervical nerves as its larger fellow.

That portion of the Superior Cervical region, which is bounded above by the External Occipital Protuberance ; below by the Superior spinous process of the Axis, in front by the inferior tubercle of the Atlas, and inferior Spinous process of the Axis, and behind by the Cervical cord, is frequently interesting and profitable, but sometimes a very troublesome region to the Veterinarian. Its anatomy calls for close study and frequent reviews if we would treat Poll Evil with the best success. Many stiff necks and enlarged Polls are standing criticisms on somebody's poor surgery.

The *Great Oblique* muscle of the head is the short, strong muscle which rotates the head around the Odontoid process of the Axis. It is covered by the Splenius and both Complexus muscles. Its inward relation is with the Atlas, Axis and Atlo-Axoid articulation. The fixed insertion is on one side of the superior spinous process of the Axis. Its movable insertion is on the transverse process of the Atlas. Blood supply is through the Prevertebral, Mastoid and Retrograde branches of the Occipital Artery and small muscular branches of the Vertebral. The second Cervical nerve furnishes its main connection with the great nerve centres. Its fibres run obliquely forward and outward.

*Small Oblique* is a little, square muscle, aponeurotic in structure, lying over the Occipito-Atloid articulation and over the origin of Occipito-Styloid and Digastricus muscles. It is in relation outwardly with tendinous and aponeurotic portions of the Small Complexus, the Splenius and Mastoido humeral muscles. Its fibres run forward and upward. They originate on the transverse process of the Atlas, and terminate at the Mastoid crest and on Styloid process and external suface of the Occipital bone. Its office is to produce a slight lateral flexion of the head on the Atlas. This muscle has the same source of blood supply as the Great Oblique; but its nerve is the first Cervical instead of second.

The Small Posterior Straight is the little, triangular, flattened muscle, which lies just over the Occipito-atloid articulation. Its under surface covers the fibrous capsule of this articulation The outer snrface relates to the Great Posterior Straight muscle, which is prismatic, and consists of two portions. It rests below on the preceding and relates above to the Great Complexus and Oblique muscles. Both straight muscles have their insertion on the Occipital bone, behind the superior insertion of the Great Complexus. The larger has its origin on the superior spinous process of the Axis and the smaller on the superior face of the Atlas. Their fibres run forward and upward. Blood is supplied through the Occipital and Vertebral arteries. Nerves, first Cervical and branch from Deep Cervical plexus. The duty of these muscles is to exend the head.

The *Great* and *Small Anterior Straight* muscles, and the *Lateral Straight* muscle will be omitted to spare your patience; and the Mastoido-humeralis briefly discussed, in conclusion, because of its functional importance and frequent involvment in Poll Evil cases. This muscle is composed of two portions, distinct in origin, but having a common insertion. The anterior, and most superficial portion, has its origin on the Mastoid crest and process of the Temporal bone. The posterior takes its origin from the transverse processes of first four Cervical vertebræ, and unites with the anterior in forming a cap over the Scapulo-humeral articulation. The common tendon, or rather aponeurosis, inserts on the anterior border of the Humeral furrow of torsion, just beneath the Deltoid imprint. This muscle runs obliquely across the cervical muscles downward and backward. The arterial supply, in the anterior Cervical region, is through the Occipital, and its main nerve is the Spinal Accessory. It is covered by the Subcutaneous muscle, Parotid gland, and Cervico Auricularis muscles It, in turn, covers portions of the Splenius, Small Complexus, both Oblique, Omo-Hyoid, Digastric, the Great Posterior Straight, Angularis, Scalenus, Small Pectoral, Both Spinatus muscles, Long Abductor of the arm and Coraco-Radial.

The arterial supply of the entire Cervical region, comes mainly through the Dorsals, Superior Cervicals, Vertebrals and Occipitals, first three being collateral branches of the Axillary arteries and the Occipitals, terminal branches of the Common Carotids. The *Dorsal* is given off opposite the second intercostal space, the *Superior Cervical* opposite the head of Second rib, and the *Vertebral* leaves the parent trunk nearly under the upper portion of the first intercostal space and passes forward *under* the transverse process of the 7th Cervical and thence into the Vertebral foramen of 6th Cervical Vertebræ. The Occipital leaves the Common Carotid trunk on a line drawn from the auricular base to the posterior portion of the Larynx. From this point it passes forward, upward, backward and forward again, like a letter "S" and goes under the transverse process of Atlas, back to the Guttural pouch, beneath the small lateral straight muscle, thence through the anterior foramen of the Atlas, where its terminal division occurs. The Dorsal and its larger branches may be readily located as follows: Draw one line to the highest point of the withers from the head of first rib. Draw a second from the same point directly upward to the Cervical cord. Intersect this second line midway and draw a third line two thirds of the distance toward the base of ear.

The *Superior Cervical* may be located, for surgical purposes, by drawing a line from the head of the first rib to highest point of withers (which point also locates the superior spinous proecsses of the 4th and 5th Dorsal vertebræ). Intersect this line miwday and draw another toward the auricular base, to a point opposite the third cervical vertebræ. Both the Dorasl and Superior cervical arteries have their origin near the median line and constantly approach the surface. The Vertebral is easily located and remembered, because after passing beneath the transverse process of 7th cervical vertebræ (which has no vertebral foramen)it simply follows the vertebral foramina of the next five cervical transverse processes and anastomoses with the Retrograde branch of the Occipital. Its only importance in this connection, lies in its superior and external branches to the muscles we have been studying.

The lymphatic vessels call for some attention, in this discussion, because of the special part they play in distributing certain pyogenic bacteria, notably the streptococci. In external portions of the body, they are arranged in two primary sets, Superficial and Deep. The Superficial, again being divided into two net works, one of very fine meshes in superficial layer of the dermis. The other includes larger vessels and is placed quite beneath the skin. The ultimate origin of these vessels is still a matter of dispute, but all agree in tracing them back to lymphatic capillaries or rootlets, described by Klein as situated in epithelial tissue. Von Recklinghausen says, in serous membranes. Even those observers who agree on connective tissue, disagree on other points, for Virchow says in plasmatic cells, while Ranvier says those cells are not cells at all, but radiating, connected spaces. Regardless of this ultimate origin question, it is that delicate plexus in the superficial derma that interests us now, and which will be made use of in discussing modes of bacterial entrance. The larger and deeper lymphatic vessels are also divided into two classes, a superficial ramifying in the tissues of the deeper aponeuroses, and a deep following the large veins, and lodged in the vasculo-nervous intermuscular sheaths. Every vessel traverses one or more lymphatic glands before emptying into the Thoracic duct or Right Lymphatic vein, those of the head, neck and front legs, are all directed toward the Prepectoral, at the chest entrance. Most of the large vessels in the region also pass through a smaller collection of glands, which lie beneath the Mastoido-humeralis and extend to insertion of the Sterno-maxillaris. These are described by anatomists under the name of Prescapular glands.

ETIOLOGY.

The next question for discussion is that of cause, and, like Darwin's first drop of proto-plasm, some points are not easily explained. In discussing this subject under the four topics, origin, bacterial—sources from whence these microbes are received, mode of entrance and factors which determine the location of the pre-fistular abscess, I wish to make incidentally the following points: That suppuration rarely or never occurs without the presence of pyogenic microbes or their products; that the initial abscess is usually the result of a local auto-infection; that fistulæ may be indirectly transmissable, due to any one or to a combination of several pyogenic microbes, and are therefore not specific; that one attack gives no immunity, but predisposes to others; that external injuries may serve to fix their location, but are not to be regarded as primary causes.

ORIGIN.—BACTERIAL.

In the early days of antisepsis, when healing without pus was first demonstrated possible, we received the old dictum "No micro-organism, no pus." And ever since those days. this question has been argued back and forth by Ogston, Fehleisen, Zuckerman, Nathan, Watson Cheyne and De Bary, who uphold this view, and Grawitz, Councilman, Uskoff, Rosenbach, Orthman and Janowski, who oppose it. Dr. Senn, in his Surgical Bacteriology, concludes that pus microbes are the essential causes of suppuration; while Fraenkel, in his text book on Bacteriology. concludes that the weight of evidence is on the other side. Some admit that suppuration may occasionally be produced by deep injections of germ-free irritants like silver nitrate, ammonia, turpentine or cadaverine; but I think the great majority of competent observers hold that the great and common cause is the presence and activity of pyogenic micro-organisms, in susceptible tissues and that certain of these organisms may be regarded as specific exciters of suppurative tissue changes.

Ogston, as early as 1881 patiently examined the contents of sixty-nine abscesses for micro-organisms, and found,

Streptococi in 17,
Staphylococci in 31.
Both in 16.

He also noted that the former followed the lymphatic channels and produced diffuse and extensive suppurations, while the Staphylococcus produced and appeared more commonly in distinct abscesses. Rosenbach carried his work farther, with better facilities, classifying and naming pyogenic microbes and adding to the list. Zuckerman tabulated 495 abscesses, showing that 71 per cent. contain d Staphyloccocus, 16 per cent. contained Streptococcus both were found in 5.5 per cent., and the other pyogenic microbes rarely. Tricomi, in 1888, reported an examination of eighty abscesses, five phlegmonous inflammations and five furruncles, and found in all one or several of the recognized pyogenic microbes. A vast amount of statistics have been published which prove beyond question that under natural conditions certain micro-organisms are always associated with the origin of pus. And it is equally plain from every day observation, that bruises or external irritants of any kind do not alone produce abscesses. True, Fehleisen, Grawitz, Scheuerlen and other investigators have produced a limited suppuration by injections of ptomaines; but this is no argument against a theory of bacterial origin.

Finally, fistulæ frequently appear in an endemic form. I have had five cases on the same pasture that developed rapidly and in close succession; in every case there was present decided elevation of temperature, loss of appetite, thirst and rapid loss of flesh. Twenty-one veteterinarians of my acquaintance have written to me in response to inquiry, that they have each seen three or more cases develop under similar conditions, in rapid succession on the same farm.

INFECTION —SOURCES.

It is not difficult to enumerate the sources from which an infection may be received; but it is frequently impossible to prove a specified origin in an individual case. Pyogenic bacteria must have a wide diffusion in nature, for they have been demonstrated in air, soil, water, and a variety of foods. There is the old question of hereditary transmission with the still unsettled points:—transmitted tissue susceptibility, or transmitted living germs, and the latter seems to be gaining ground, with plenty of clinical evidence in its favor. Lebedeff reports a case where he found Streptococci in various tissues of a child prematurely born, of a mother who recently had erysipelas. Sangalli reported the finding of Bacillus Anthracis in the blood of a fœtus, whose mother had died of carbuncle. Ahlfeld and Marchand, together, reported two autopsies, which showed that a child had died four days after birth from anthrax, and the mother died eight hours after delivery from the same cause. Netter, Bollinger, Strauss, Koubassoff and Levy have presented similar cases of pathogenic germs acquired in utero. Johne, in 1890, published a calf case more interesting to Iowa veterinarians than our celebrated Jones county calf case to Iowa lawyers. In this case an eight months fœtus was taken from the body of a tubercular cow. The fœtal lungs and liver both contained tubercles and bacilli of tuberculosis. Levy reports a case wherein the pneumonia diplococcus of Frankel and Weichselbaum played a similar part. In view of all this evidence, we can hardly doubt the possibility of actual germ inheritance and especially since Nepveu has shown, in two cases, that pyogenic organisms may remain dormant or encysted for years, awaiting favorable conditions for development, and then cause profound disturbance. Gussenbauer, Rinne and Senn have all reported similar cases, which clearly prove that apparent pathological conditions do not necessarily follow immediately the introduction of the microbes, and which prove also, that the absence of such conditions does not demonstrate an abscence of pyogenic organisms. To sum up this discussion of source, we can easily say that these troublesome micro-organisms are derived from the soil, air, food, and maternal circulation.

### MODES OF ENTRANCE.

That the actual transmission of living micro-organisms from mother to fœtus *in utero* is possible, and probably frequent, has been fairly established by competent observers, whose notes and opinions have already been given. Fraenkel, in explaining the sources of errors which may interfere with inoculation experiments, says :—"There are three ways in which micro-organisms usually penetrate into our bodies : First, from the surface of the skin, generally after it has been injured in some way. It does not, however, indeed always require such special door of entry (i.e. abrasion). Second, the digestive canal, into which the bacteria pass along with the food. Many, it is true, cannot pass through the stomach in their usual form, being destroyed by the action of its acid contents. Other kinds are less sensitive, and when spores are present, or when disease has altered the character of the digestive fluid, and weakened its bacteria-killing power, there is no further obstacle to the passage of the parasites. Third, the respiratory organs can afford entrance to the bacteria."

Zuckerman, in summing up his experiments on suppuration, says plainly that pus-microbes can enter the body through the skin intestinal mucous membrane, or by way of the respiratory organs ; but the most frequent entrance is through the skin. Longard, in discussing certain superficial abscesses, declares, as his opinion, that they are caused by pus-microbes, which gained access through the sweat glands, and gives as his reason for the latter proposition, that these certain microbes were found in abundance on the inner surface of the *membrana propria* of these glands where they remained harmless till they penetrated to the under-lying connective tissue ; and why may not this be correct ? The continuous lumen of the coiled tube which constitutes a sweat gland, and sudoriferous canal which connects the gland with the body surface, is covered internally with a very thin delicate membrane. This membrane is covered by two or three layers of polyhedral, epithelial cells, and over these lies the *membrana propria*. Now, if those pyogenic microbes had reached the *membrana propria*, why may they not reach the underlying connective tissue of the corium, or even the subcutaneous tissue ? Each gland has an afferent arteriole from which is formed a delicate plexus of capillaries, surrounding the gland tube. Thus are furnished the necessary conditions for receiving and distributing these pyogenic organisms.

Kraske has made a special study of acute Osteomyelitis, and reached a positive conclusion that infection may take place through abrasions of the skin, through the respiratory organs, or intestinal mucous membrane ; but most commonly through the abraded skin. In one case he traces an acute attack to infection from a furuncle of the lip. Garre, Braunschweig, Schimmelbusch, Roth, and others have all demonstrated that healthy skin is not impenetrable to living micro-organisms. The experiments of Buchner show positively that the respiratory organs may furnish an entrance to a variety of microscopic germs. There can be no doubt that abrasions of either the respiratory or intestinal mucous membranes or of the skin, greatly facilitate their entrance ; but it is equally certain that their entrance is possible and common when no such conditions are offered.

### LOCATING FACTORS.

After discussing the bacterial origin ; the sources from which inflection may be drawn and manner of entrance, there yet remains the query ; what determines the location of the pre-fistular abscess ? It has long been a matter of dispute, whether pathogenic microbes exist in healthy animal tissues. I think the veiw gaining general support that such conditions are possible ; but that these same microbes do not exhibit their pathogenic properties, so long as they remain in circulating blood and every body-tissue remains healthy., Then, to furnish the requisite conditions, under which these pyogenic microbes may exhibit their pathogenic functions, some susceptible tissue must become injured or diseased. The blood or lymphatic fluid must be checked in its flow at that point, thrombosis be formed and the microbes be permitted to locate and multiply. In other words, to locate pre-fistular abscess, there must be furnished a *locus minoris resistentiæ*, which Senn defines as being an area of lessened resistance due to a tissue injury, which so changes the tissue that pathogenic microbes, previously present in the circulation, become arrested, and find favorable conditions for muliplication. Huber asserts that several infectious diseases as Tubercular Ostitis and Arthritis, Osteomyelitis and pyæmia may follow trauma in the absence of any possible local external infection His experiments on rabbits with Anthrax bacilli are well worthy of study. He produced an inflammation of one ear by applying croton oil, leaving the other untouched. He then injected a pure culture of Bacillus Anthracis and compared results of the two ears. His conclusions are that " The bacillus of Anthrax finds in a soil prepared by an inflammation, induced by croton oil, a locus minoris resistentiæ, which presents more favorable conditions for its growth, than tissues in other parts of the body." His conclusions may be summarized as follows: " Localization of pre-existing micro-organisms in tissues prepared by injury or disease, takes place, provided that the necessary conditions for their growth are present."—*Senn.*

But recent tissue lessons are not the only factors which may serve to produce such an area of lessened tissue resistance. The presence of old pathological products, exposure, feeble performance of any organic function, and a variety of general and illy defined factors seem to produce conditions favorable to local infection. The regions which fistulæ usually select are such as are liable to receive blows and bruises, given by angry attendants; received in passing under low sheds, and through low doors, or while rolling. Again, the soft tissues of these parts are mainly connective, and connective tissue areas are very prone to chronic suppurative processes and healing, because circulation is slow.

Such then are the factors which serve to locate suppurative processes.

### NEED OF CONTINUED RESEARCH IN THIS FIELD.

There yet remain many questions regarding the history and work of pyogenic microbes for bacteriologists and chemists to answer. We all wish to know to what depth beyond the surface of a pyogenic membrane do these pus producing germs reach. Why does pus "burrow"? Is it by pressure and absorption? Is it due to a peptonizing activity of bacterial waste products, or it is due to some mysterious part played by the pyogenic microbes in their effect upon protoplasmic cell contents? We do not know so much as we would wish regarding the metastatic tendency of suppurative processes. Fistulæ sometimes heal rapidly and abscesses develope in the Atlo-Axoid region.

There are some strange features in such cases. Why do not other domestic animals than solipeds commonly develope fistulæ? Is it a question of tissue resistence, anatomical peculiarity, or a difference in histological elements? The chemist might give us a non-toxic, diffusible antiseptic, that will pass through the circulation and be excreted unchanged and capable all the while, of destroying pathogenic microbes. There is a mysterious part played by certain portions of the lymphatic system that we could wish made plain. We do not even feel certain that we know why fistulæ occur more frequently in the Superior Cervical than in the Gluteal or Dorsal region.

TREATMENT.

There are many annoying features which appear in the treatment of these cases, which may be well understood and yet difficult to handle; for example, the well-known tendency to recur after indefinite periods of apparent soundness, just such cases as a physician must deal with in a latent and local tuberculosis, which may, under certain conditions, become suddenly general and active. Or like the case reported by Gussenbauer, in which an apparently strong, healthy young man developed a case of Lymphangitis, and Lymph-adenitis of left arm, followed by a circumscribed gangrene of skin and flexor tendons. Lymphatic glands did not suppurate, but enlarged. After eight months of good health a large acute abscess in left axilla, which convinced Gussenbauer that pyogenic organisms had remained in those glands latent for eight months. Nepveau reports two similar cases, in which the latent periods were much longer.

Internal treatment, consisting of alteratives and tonics may sometimes be used with advantage. Twenty-nine practitioners out of the forty-nine with whom I have corresponded on this subject, advise more common use of such treatment. Generous food and tonics are always in order if patient is unthrifty.

The conditions which experience has taught me are necessary to successful treatment of fistulæ are briefly :—

(*a*) Free escape of pus to carry out microbes and ptomaines.

(*b*) Destruction of the pyogenic membrane, lining old sinuses and cavities.

(*c*) Destruction of accessible pyogenic microbes.

(*d*) Removal of foreign substances as splinters of wood, or necrosed bone.

(*e*) Establishment of an active granulating process.

The first I strive to attain at any cost. I accomplish such drainage by the knife, trochar and canula, setons of cloth, or rubber drainage tubing and a surgeon's large pump. We sometimes need a seton that will remain in place for a long period and not decay. Horse-hair, rubber tubing, shoe-maker's linen thread or leather will answer this condition perfectly. I have never tried to trephine the scapula, but believe it can be done with advantage in those cases where a sinus runs down into the subscapular region; at least I shall certainly try the operation when the first favorable case presents. In a few cases bottom drainage seems impossible. The only satisfactory substitute with me has been hydrogen peroxide. The next condition is secured by digestant solutions, by curette or by severe, but self-limiting caustics like pure carbolic acid, or concentrated alcoholic solution of bichloride. Copper, zinc and the other metallic salts have not proven so satisfactory to me. Diluted nitric acid has given me good results, but my preference is 95 per cent. carbolic acid, and the sharp uterine curette (human). Dilute bichloride, dilute carbolic, and peroxide have proven the most practical antiseptics. The only way to get rid of foreign substances is by bold surgery. The production of active, healthy granulations sometimes calls for all the ingenuity a surgeon can muster. Turpentine, ammonia, common salt solutions, and diluted white liniment have all given good results; but the practitioner must be cautious in using them. Long continued and repeated irrigations with hot dilute antiseptic solutions and even hot water, or hot and cold water quickly alternating in a steady current, will frequently transform a hopeless old chronic into a very interesting patient. A blister covering a large surface, and severe, will sometimes bring about similar results; but is painful, and frequently needless. I only use it in those cases where the discharge nearly ceases, but is annoyingly persistent.

Through the kindness of Iowa Veterinarians, I have accurate reports on 350 cases of *fistulous* withers, 133 *poll evils*, 72 cases where both appeared on same patient, and 103 fistulous sores in other regions. And in addition the 20 cases of fistulous withers given under experimental work.

### RESULTS, AND TIME REQUIRED.

Of the 350 cases of *fistulous withers*, 87.5 per cent. healed under various forms of treatment. The shortest time required 17 days, longest 126. Average of all cases reported, 53 days. Of the 133

cases of *poll evil* 84.9 per cent. healed with similar treatments, average time required, 42 days. Of the 72 cases where both appeared on same patient, 77.7 per cent. healed ; I cannot give accurate figures on time required, but evidently it exceeded that of either *fistula* or *poll evil.* Of the 103 cases of fistula sores in other regions, 92 per cent. healed rapidly. These appeared as follows: scrotal, six ; mammary gland, five ; ear, eleven ; superior maxilla, eight ; inferior maxilla, seven; rectal, six; dorsal region, nine; mid-cervical, two ; costal, nine ; brachial, two ; tibial, three ; patella, one ; parotid, one ; tarsal, two ; flank, six ; vagina, one ; scapula, one ; trachea, one ; pectoral, eight ; Steno's duct, two ; femoral, five ; sacral, one.

The records of thirteen successive cases of fistulous withers, which occurred in my own practice during a certain period, and which were treated according to the general method herein given show 92 per cent. cured, average period of treatment 46.5 days.

### PROGNOSIS.

A study of these cases and the results reported for each shows plainly that the prognosis does not depend so much on the shape of the head, or style of withers, as frequently stated, as on the location of prefistular abscess and subsequent sinuses.

Next in importance is the physical condition of patient.

### EXPERIMENTAL TREATMENT.

My own work during the past year has been such that I could not personally carry any continued series of experiments, and so I had to recourse to friends interested in the same work, who tried for me a certain line of treatment, and reported results. I have no copy of the letter which was sent to them, but taken from memory and in general it was as follows : The separate items used in the order given once daily ; abundant bottom drainage, irrigate thoroughly with 3 per cent. carbolic acid, or 1-1500 bichloride, rinse out the sinuses with pure water, plug the inferior opening, and fill sinuses with a warm aqueous solution of hydrochloric acid 2 per cent., and any reliable scale pepsin (*e.g.* Dike's or Fairchild's) 10 per cent., leave undisturbed three hours, remove plug and irrigate again with antiseptic solution, to be continued as seems necessary to secure the desired condition.

I have records of twenty cases so treated. These show nineteen, or 95.6 per cent. cured, in an average of 36.2 days. The details must, of course, be varied to suit individual cases, and even for differing stages in the same case. Larger experience may teach that less pepsin is necessary, and that more or less of the acid will be more satisfactory.

Those who have tried this method agree that results are more rapid and satisfactory than with the older lines of treatment. There seem to be two objections: First, expensiveness; second, patient must be under the immediate care of operator. The last can have but little weight, because that is where every case of fistula should be.

### CASES.

I have full notes on the history and treatment of thirteen cases, with criticisms on my own work, which I had hoped to present, but this paper has already reached such length that I will give only a few of the most interesting and difficult.

Case A.—Bay horse, ten years old, 1,100 pounds and thin in flesh, had been treated for five years by empirics and considered hopeless. An examination April 5th, 1889, developed four sinuses with common outlet, located as follows: the first sinus was four inches deep, with an outlet two inches anterior to the cervical angles of the scapulæ at the median line. Probe passed straight down between lamellar portion of cervical ligament on one side, and Rhomboideus, Angularis, Great and small Complexus muscles on the other. The second was located between the same structures, but had its direction backward and downward to anterior border of scapula—two and one-half inches long. The third sinus, five inches long, extended from the common outlet downward, forward and to the right, passing through the Complexus Magnus and Splenius, and terminated between the Cervical Trapezius. Fourth, ten inches long, extended from the outlet, downward, forward, and toward the left, and terminated beneath the Cuticularis Colli. The treatment of this case was quite simple. Sinuses (3) and (4) were given inferior drainage by very large incisions at the lowest point. Sinuses (1) and (2) were drained by large setons passed from similar points to the surface in a downward direction. These sinuses were washed out daily with 1 : 3000 bi-chloride solution. The setons were left in place two weeks, and daily moved up and down. Each week the patient was brought to town for examination and treatment. The treatment usually consisted of a thorough flushing with cold water, after which each sinus received a small injection of a mild white liniment. August 10th, an examination showed a sound horse, and there has been no recurrence during three and a half years. The outlet and both drainage incisions were re-opened by the knife every two or three weeks. Criticisms—(*a*) Later experience has taught me that quicker results would have been obtained with less washing and irrigation. (*b*) The setons would have been left in place at least a month. (*c*) I should have insisted on having the horse under daily observation.

Case " D" Iron Grey filly, two years old, never had a collar on, sheds and doors all high, running in a woodland pasture with case "C." Examination Aug. 20th, 1890, showed merely a large hygroma on left side, two inches in front of Cervical angle of Scapula. Contents thin, serum-like fluid, without offensive odor. Free incisions were made above and below; the cavity, cleansed and packed with bichloride cotton. Somewhere at this point I made an error, for the case was on hand, uncured all fall and winter. There was either an early developed and undiscovered sinus, down beside the Cervical lamina, or a piece of cotton was left in the large cavity. In this case I expected a rapid recovery and gave very little treatment during the fall; was away from my practice all winter, and on my return in April found case" D" had developed into an old chronic and was discharging freely from several openings. One sinus passed straight down beside the laminar portion of Cervical ligament, five inches, and just in front of the anterior Scapular borders. There were several short and superficial sinuses which I could lay open from top to bottom; but that deep sinus was a puzzle, owing to its peculiar location. The treatment decided upon was a long seton of rubber drainage tubing, passed from the outlet diagonally downward, forward and to the right a distance through and between muscles of fourteen inches. The shallow sinuses closed in 12 days. The long sinus and drainage tube were flushed out occasionlly with mild antiseptie solutions. The tube was drawn down and clipped off an inch every other day. This process was required about five weeks and gave good results, for the sinus closed above as the tube was withdrawn. Notes show case " D" to have been dismissed June 1st, 1891. Sept. 23d, a small abscess was formed on right side, next the Cervial cord and near the old outlet, which soon ruptured. The opening was slightly enlarged and the cavity with its short sinus were filled with ninety-five per cent. carbolic acid. There was profuse discharge for a week, then rapid and permanent healing. Oct. 15th, " D" was sound again and has so remained ever since. Here was a case of dormant pyogenic microbes, becoming active after a *locus minoris resistentiae* had been formed perhaps by bruise or other tissue injury. Criticisms: (a) A lesson-teaching incident occured when the drainage tube had been partly removed. A sudden swelling and stiffness of the front leg on that side occurred when the drainage tube had been partly removed. Evidently a septic inflammation. Vigorous examination by the probe discovered and opened a large pus cavity at one side of the artificial sinus. My only explanation is that the broad bladed seton needle passed through the body of the small Complexus parellel with the muscle fibres, and when the seton was drawn below they closed and healed rapidly confining pus above. (b) I made a very grave mistake in opening the cyst as long as there was any possibility of absorbtion.

(c) I should have made a more careful internal examination of the abscess in the first place and taken nothing for granted. I should also have removed the cotton personally.

(d) We make a mistake always when attempting to treat a fistula at long range.

Cases "E" and "F" were fully as interesting and difficult. My notes show "B" and "G" to have been very easy and rapidly successful. The next five cases appear among horses in the same pasture, all but one of which had poll evil or fistula, and some, both, during one spring and the early summer. I was first called July 1st, 1891. The history of this outbreak is rather meagre. I was only able to learn that case "I" was a brown mare, seven years old, previously in good health and flesh, exhibited a decided swelling over the Atlo-axoid region about March 20, 1890. This swelling subsided before March, 1891. Another swelling developed rapidly, soon ruptured and discharged very profusely over the pastures and feed boxes. Some lung difficulty, probably septic in origin developed, followed by a nasal discharge and the patient died in June of the same year. Other cases followed rapidly.

Case "J" g. m., 1400 lbs., six years old, very fat at first, exhibited a double poll evil and a fistula on right side of withers, lost flesh very rapidly, and on my arrival was found to be very thin and stiff.

Case "K," dark grey mare, five years old, 1100 lbs., also in good flesh at first. A double poll evil began to develop June 16th, 1891. Very large at the time of examination, condition fair, appetite poor; in all these cases they all showed 1.5 to 2.5 degrees elevations of temperature.

Case "L," dark grey, three years, 1200 lbs., began to swell on both sides of withers June 29th, 1891, in good flesh Jan. 1st; appetite good and no discharge.

Sodium sulphite was alternated with arsenic, one dose of each daily. In none of these cases was the local treatment difficult to manage, and consisted of self-drainage by free incisions, and a limited amount of antiseptic flushing by syringe, with weekly stimulations. All recovered except "I," which had died in June.

Notes :—Cases "C" and "D" were two unbroken colts in the same pasture. "C" developed and discharged two months before anything wrong was noticed with "D." These colts did not even have access to a shed. The cases I, J, K, and L had access only to a first class barn and shed, with wide, high doors. Nothing of interest could be discovered in the pasture except an artificial pond, which contained stagnant water, and out of which the horses must have drank for there was no other water accessible. It seems plain to me that such suppurative processes may be indirectly transmissable and more than local in effect.

A proposition which Chauveau, Orth, Rosenbach, and Wissokowitsch have proven correct regarding source, locating factors and results of pus microbes in other conditions, and which Huber proved true for Bacillus Anthracis, and which Volkman, Schueller, Szuman and Verchere have proven for Tuberculosis, is certainly worthy of consideration as applied to the pathological condition under discussion.

Finally I wish to express my indebtedness to the printed works of Chauveau, Crookshank, Frankel, Vaughn, Steinberg, to the Veterinarians of Iowa, who aided me by accurate reports, and especially to Drs. Edwards, of Iowa City, Hammond, Le Mars, Williams of Manning, and Derwent of Marshalltown, who conducted the pepsin experiments.

---

### ARTIFICIAL FECUNDATION.

Mr. Everitt Millias, the investigator of distemper, has successfully performed artificial fecundation of bitches by collecting the male elements in a vessel, which was floating in another vessel kept up to the temperature of 100 deg. Fahr. with hot water—the water being in the outermost cup and floating the reservoir containing the vital germs—and injecting the same by means of an ordinary human catheter fixed to an ear syringe. The bitch so treated had *two puppies.* On another occasion Mr. Millias fertilized no less than three bitches on the same day from a single discharge of a dog. It is highly interesting and may be useful, to know that this operation is possible, for there are many dogs who, in full possession of all their reproductive faculties, show an utter disinclination to exercise them.*—*Abstract from the Veterinary Record.*

* The fecundation of animals suffering from imperfections or obstructions in the generative passages seems most inadvisable. [Ed.]

